# Pheno2Geno - High-throughput generation of genetic markers and maps from molecular phenotypes for crosses between inbred strains

**DOI:** 10.1186/s12859-015-0475-6

**Published:** 2015-02-19

**Authors:** Konrad Zych, Yang Li, Joeri K van der Velde, Ronny VL Joosen, Wilco Ligterink, Ritsert C Jansen, Danny Arends

**Affiliations:** University of Groningen, Groningen Bioinformatics Centre, Nijenborgh 7, Groningen, 9747 AG The Netherlands; University of Groningen, University Medical Center Groningen, Genomics Coordination Center, PO Box 30001, Groningen, 9700 RB The Netherlands; University of Groningen, University Medical Center Groningen, Department of Genetics, PO Box 30001, Groningen, 9700 RB The Netherlands; Wageningen University, Wageningen Seed Lab, Droevendaalsesteeg 1, Wageningen, The Netherlands; Jagiellonian University, Faculty of Biochemistry, Biophysics and Biotechnology, Gronostajowa Street 7, Krakow, 30-387 Poland

**Keywords:** Quantitative genetics, Computational genomics, Genotyping, QTL mapping, Recombination, SNPs, Tiling arrays

## Abstract

**Background:**

Genetic markers and maps are instrumental in quantitative trait locus (QTL) mapping in segregating populations. The resolution of QTL localization depends on the number of informative recombinations in the population and how well they are tagged by markers. Larger populations and denser marker maps are better for detecting and locating QTLs. Marker maps that are initially too sparse can be saturated or derived *de novo* from high-throughput omics data, (e.g. gene expression, protein or metabolite abundance). If these molecular phenotypes are affected by genetic variation due to a major QTL they will show a clear multimodal distribution. Using this information, phenotypes can be converted into genetic markers.

**Results:**

The Pheno2Geno tool uses mixture modeling to select phenotypes and transform them into genetic markers suitable for construction and/or saturation of a genetic map. Pheno2Geno excludes candidate genetic markers that show evidence for multiple possibly epistatically interacting QTL and/or interaction with the environment, in order to provide a set of robust markers for follow-up QTL mapping.

We demonstrate the use of Pheno2Geno on gene expression data of 370,000 probes in 148 *A. thaliana* recombinant inbred lines. Pheno2Geno is able to saturate the existing genetic map, decreasing the average distance between markers from 7.1 cM to 0.89 cM, close to the theoretical limit of 0.68 cM (with 148 individuals we expect a recombination every 100/148=0.68 cM); this pinpointed almost all of the informative recombinations in the population.

**Conclusion:**

The Pheno2Geno package makes use of genome-wide molecular profiling and provides a tool for high-throughput *de novo* map construction and saturation of existing genetic maps. Processing of the showcase dataset takes less than 30 minutes on an average desktop PC. Pheno2Geno improves QTL mapping results at no additional laboratory cost and with minimum computational effort. Its results are formatted for direct use in R/qtl, the leading R package for QTL studies. Pheno2Geno is freely available on CRAN under “GNU GPL v3”. The Pheno2Geno package as well as the tutorial can also be found at: http://pheno2geno.nl.

**Electronic supplementary material:**

The online version of this article (doi:10.1186/s12859-015-0475-6) contains supplementary material, which is available to authorized users.

## Background

Quantitative trait locus (QTL) mapping is a powerful approach used in population analysis to link genetic variation with phenotypic variation [1]. It requires polymorphic genetic markers positioned on a genetic map. The denser the genetic map, the lower the chance of missing true QTLs. Furthermore, more markers (i.e. greater density) yield a more accurate overview of informative recombinations. Theoretical limit of resolution depends on the size of the genome, size of the population and type of the cross.

Phenotypes showing a dichotomous 0/1 distribution with approximately equal proportions in, for example, a recombinant inbred line (RIL) population can be used as genetic markers: genotypes can be called by connecting the 0/1 to the parental strains A/B. Such markers can then be used for *de novo* construction of the genetic map or for saturation of a known genetic map [2,3]. A simple approach using mean parental expression to split dichotomous phenotypes into A/B categories works for large datasets (*n*>22,000 markers [2]).

Continuous (non-dichotomous) phenotypes can also be used as markers if they show a major QTL, since a major QTL will cause the phenotype to show a clear multimodal distribution to which a mixture model can be fitted [4,5]. Posterior probabilities derived from mixture modeling are used for genotype calling, and such approaches have been used to derive genetic markers for up to 1,200 molecular phenotypes [6].

In order to construct or improve genetic maps using high-throughput molecular markers, we scale up the mixture model approach for non-dichotomous phenotypes such as gene expression data. High performance mixture models have been used to perform genotype calling in SNP arrays [7-9]. The new tool we have developed, Pheno2Geno, allows the use of comparable amounts of expression data either to saturate genetic maps, or to derive them *de novo*.

## Implementation

Pheno2Geno provides the following functionalities to saturate and generate genetic maps.

**1) Preprocessing of the data**: Pheno2Geno offers a selection of data transformation functions (log, sqrt, reciprocal, probit and logit). Gene expression data measured using microarrays are generally log or square root transformed before further analysis [5,6,10]. Details about which method to select can be found in the manual of the Pheno2Geno package.

**2) Analysis of parents of a segregating population**: When parental data are available, Pheno2Geno uses a *t*-test to select molecular phenotypes showing significant differences between parental strains of a segregating population. Such an approach is only possible when parental data are replicated at least three times. This reduces the computational load by restricting the analysis to molecular phenotypes that show differential expression between parents.

**3) Analysis of a segregating population**: Phenotypes with a major QTL will show clear multimodal distributions in a segregating population. Pheno2Geno fits a mixture model to the phenotype distribution [4,5,11]. Phenotypes showing a multi modal distribution are selected as candidate markers by a user defined significance threshold. Pheno2Geno then tests these candidate markers to predict if mixing proportions are close to the expected segregation frequency, e.g. 1:1 for a bimodal distribution of two homozygous classes in an F2-derived RIL or 1:2:1 for a trimodal distribution of two homozygous and one heterozygous class in an F2 cross. The deviation allowed from this expected segregation frequency can then be set by the user.

**4) Assigning genotypes**: For each of the candidate markers, the posterior probabilities of belonging to each of the component distributions in the mixture are calculated [11,12]. Using these posterior probabilities, the continuous phenotype values are converted into discrete data (e.g. 0 or 1 for RILs; 0, 1 or 2 for F2). If the posterior probability of a specific marker-individual combination is lower than a user-specified threshold, a missing value (*) or partly informative value (e.g. not 0, but homozygous 2 or heterozygous 1) is assigned to avoid introducing genotyping errors. If parental data are available, these can be given a parental origin label (A or B for RILs, A, H or B for F2). If parental data are not available, mixture-model-based scores cannot be converted into parental origin labels. In the case of RILs, Pheno2Geno is able to solve this problem by forming twice as many linkage groups compared to the expected number of chromosomes. Pheno2Geno then looks for the combination of two linkage groups that show strong negative correlation. If a pair of negatively correlated linkage groups is found, genotypes from one of these linkage groups are inverted. After which the two linkage groups are merged into a single chromosome.

**5)*De novo* construction of genetic maps**: If no genetic map is available, Pheno2Geno can be used to create an initial ’skeleton’ map. This is produced using very strict settings in the mixture model analysis to obtain a limited number of highly trustworthy markers. These candidate markers are assigned to linkage groups using the R/qtl function *‘formLinkageGroups’*. Additional information provided by the user can be used in this step, for example, known physical and genetic positions. The package then uses the known physical positions to assign physical chromosome IDs to linkage groups and to determine the correct orientation of the chromosomes. Pheno2Geno subsequently orders all the markers inside a linkage group using the R/qtl *‘orderMarkers’* function. Finally, the skeleton map is saturated to improve resolution, as described below.

**6) Saturation of a known map**: Pheno2Geno performs interval mapping of candidate markers on the original map using the R/qtl *‘scanone’* function. When a candidate marker has a single QTL peak, it is placed at this position. The map is then re-estimated using the R/qtl *‘est.map’* function, followed by removal of duplicate candidate markers and markers located at the position of a known marker. West et al. [13] emphasized that creating genetic markers from gene expression data is seriously hampered by the presence of environmental variation and multiple, possibly interacting, QTLs (epistasis). Pheno2Geno uses R/qtl to test if candidate markers are affected by multiple QTLs or pairwise interactions. This is done by performing a two-dimensional genome scan with a two-QTL model. Additionally, if the data was measured in multiple environments, potential environmental interactions are tested. The user then decides whether affected candidate markers are flagged or removed from further analysis.

**7) Detection of errors**: After saturation or *de novo* construction of a genetic map, Pheno2Geno can detect and correct genotyping errors, e.g. double recombinations, missing data or semi-informative markers. Missing genotype data can be imputed using the R/qtl function *‘fill.geno’*, which allows users to perform genome scans by marker regression without having to drop individuals with missing genotype data. However, this should be done with care as the resulting genotype data will be dubious when a large number of missing genotypes have been imputed. Furthermore, when saturating a known map with available genotype data, Pheno2Geno can detect sample mix-ups in the original data using *‘R/lineup’*, which is a part of the R/qtl toolset. External tools, such as MixupMapper [14] can also be used to detect and correct the original genotype data.

Genetic maps created by Pheno2Geno can easily be used for QTL mapping. The package provides output structures compatible with R/qtl, the leading R package for QTL analysis in experimental crosses [15,16]. Pheno2Geno allows users to explore and compare the resulting maps with their favorite genome browser. Maps can be saved as a GFF (General Feature Format) which is supported by most genome browsers.

## Results

To test Pheno2Geno, we analyzed a population with a sparse genetic map. The original Amplified Fragment Length Polymorphism (AFLP) map was created using a population of 420 RILs derived from a cross between *Arabidopsis thaliana* Bayreuth (Bay-0) x Shahdara (Sha). The original map contained 69 AFLP markers at an average map distance of 7.1 cM [17].

Our dataset consists of 148 RILs from the cross, which were assigned to four different conditions using the designGG package [18]. Each of the parents was measured twice per condition. Gene expression per line was measured using tiling arrays (370,000 oligonucleotide probes per array).

In total, 10,801 phenotypes (the input set) were detected as being differentially expressed between parents (*P*<0.01), and we did not correct for multiple testing, because the input set was small enough to be handled efficiently by Pheno2Geno. Mixture modeling identified 1,230 selected phenotypes having approximately a 1:1 segregation ratio. Pheno2Geno removed 267 phenotypes as potential markers showing QTL by environment interaction (*LOD*>=7.5), 7 markers with multiple QTLs (*LOD*>15), 279 candidate markers showing no QTL, and 77 candidate markers that appeared to show pairwise epistatic interactions (*LOD*>=7.5).

**Figure 1:**
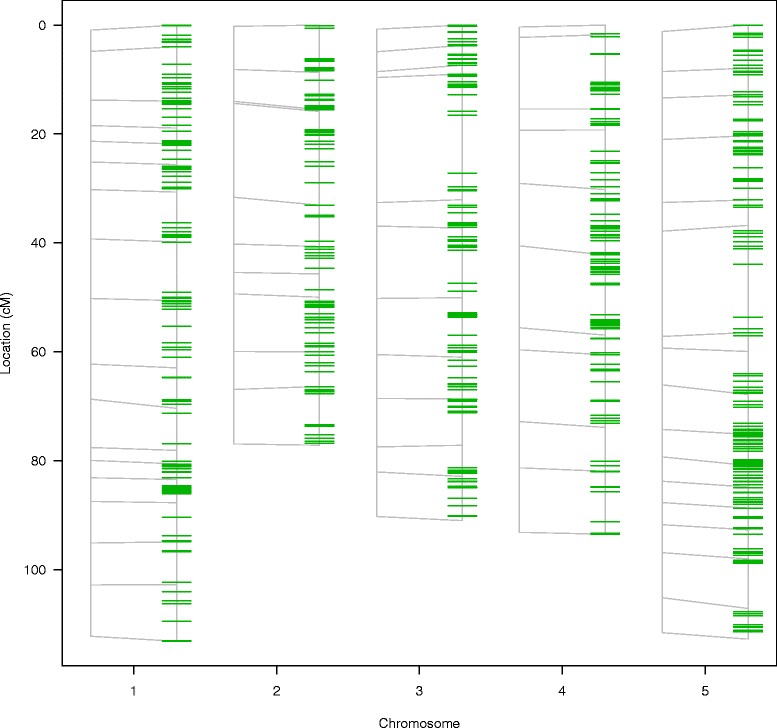
**Saturation results.** A map comparison plot generated using the R/qtl function *’plot.map’* [15,16]. For each of the chromosomes, the original map (left) and the saturated map (right) are plotted. Lines are drawn to connect markers. Markers that exist in one map but not the other are indicated by short line segments. Before plotting, both maps were re-estimated using the R/qtl function *’est.map’*. The original map consisted of five chromosomes and 69 markers at an average distance of 7.1 cM. The saturated map consists of the original 69 markers plus 497 expression-based markers at an average marker distance of 0.89 cM.

Using the remaining 600 candidate markers the original map was saturated and 103 co-localizing markers were removed. This resulted in 497 new gene-expression-based markers (8 times the original number). The original and saturated maps were re-estimated using the Kosambi map function using the R/qtl function *‘est.map’*. Map expansion was observed for chromosomes 1 and 5, increasing the total map length from 480.7 to 501.5 cM (Figure 1). Saturation resulted in a decrease of the average map distance from 7.1 cM to 0.89 cM, while saturation of the *A. thaliana* Bay-0 x Sha map led to more than a sevenfold improvement in marker density. This means that for studies, in which molecular phenotypes were already measured, this improvement can be achieved without any additional lab costs.

Resolution of a genetic map is limited by the size of the population from which the map is derived. A distance of 1 cM is equal to 1 recombination per 100 individuals. Our sample size of 148 individuals implies that Pheno2Geno could obtain, a theoretical resolution as high as 0.68 cM between markers. The resolution of the Pheno2Geno saturated map for *A.thaliana* is 0.89 cM, which is very close to this theoretical limit.

A *de novo* reconstruction using only gene expression data (ignoring the original markers and map) resulted in a skeleton map containing 227 markers with an average distance of 2.2 cM. This skeleton map had a length of 499.4 cM, and could be saturated again using less strict parameters.

**Figure 2:**
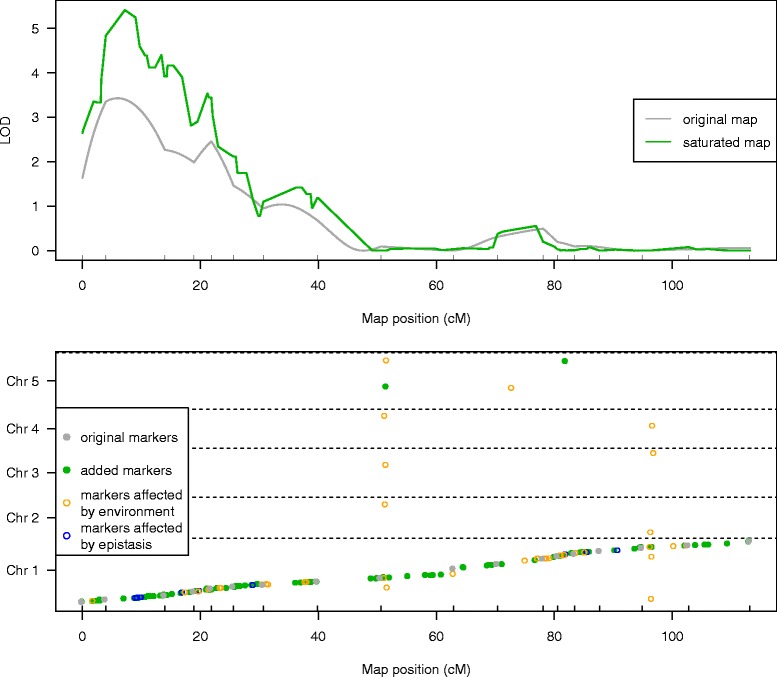
**Comparison of QTL profiles.** **Top** Results of single-marker QTL mapping of a classical phenotype (y-axis) on the original (gray line), and the saturated map (green line). Only chromosome 1 is shown. **X-axis** - positions of the original markers on the genetic map. **Bottom** Positions of probes used during marker generation on chromosome 1. **Gray dots** - show positions of the original markers on the physical map. **Colored dots and circles** - show candidate markers detected by Pheno2Geno. **Orange circles** - show candidate markers removed because they showed significant environmental influence. **Blue circles** - show candidate markers removed because they showed an epistatic interaction with other genetic markers. **Green dots** - show markers used for saturation of the original map. The final saturated map consists of all the green and gray dots. The locations of the new markers on the old map are shown here so that maps are aligned for better clarity.

Additionally, we performed QTL mapping of our published classical phenotype dataset [19] onto the saturated map. As an example we show the QTL profile of the trait “Time when 50% of seeds have germinated under 100 mM NaCl” (Figure 2). Re-mapping the entire dataset of classical phenotypes onto this new map shows an increase in QTL likelihood for 56% of the previously detected QTLs. Additionally, 29 new QTLs were detected on the saturated map, increasing the number of QTLs from 213 to 242. These QTLs have LOD scores close to the significance threshold when mapped onto the original map with LOD scores between 3.4 and 5.

**Figure 3:**
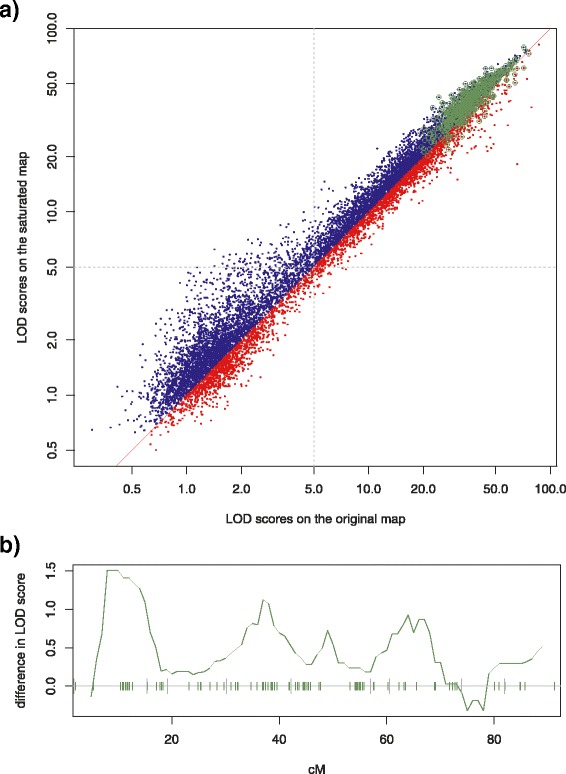
**Comparison of QTL detection power.** **a)** LOD scores on the original and the saturated map. QTL mapping was performed on all 10,801 tiling array probes showing differential expression between parents (*p*<0.01 Student t-test) using the original and saturated maps. 5,837 out of 10,801 probes show a QTL with a *L* *O* *D*>5 on the original map. **Blue dots** - represent 3,943 probes (67.6%) that show an increased LOD score on the new saturated map. Moreover, 210 new QTLs were detected on the saturated map. **Red dots** - probes showing a decrease in LOD score on the saturated map. **Green circles** - are probes used to saturate the map. **b)** Changing LOD scores. For each of the phenotypes the top QTL peak was selected. If the peaks measured on the original and saturated maps shared a location, then the difference between the LOD scores was calculated. **Solid green line** - shows median of differences between QTL peaks from chromosome 4, calculated inside a sliding 10 cM window stepped across the chromosome with a step of 1 cM. For each of the windows the value was plotted in the middle of the compartment (thus no value for the first and the last 5 cM). Ticks on the x-axis show the position of the markers: **tall gray ticks** - show original markers; **short green ticks** - show markers selected by Pheno2Geno. Only one region, in which no new markers were added (75-80 cM), does not show an increase in power.

Finally, a QTL mapping of all the gene expression probes showing differential expression between parents (10,801 probes) was performed, and 5,837 probes had a significant (*LOD*>5) QTL on the original map. Out of these, 3,943 probes (67.6%) showed an increase in QTL likelihood on the saturated map (Figure 3) and an additional 210 new significant QTLs were detected on the saturated map.

## Conclusions and discussion

We have developed Pheno2Geno as a generic software package for generating genetic markers and maps from high-throughput molecular phenotypes. The package works for any inbred diploid population, e.g. backcross, F2 intercross and recombinant inbred lines. Pheno2Geno has four important features:

**1) Big data computation.** Pheno2Geno can process large volumes of different kinds of molecular phenotypes [20] including gene expression, protein- and metabolite abundance. The memory requirements of the algorithm are reduced by reading in and processing files in chunks rather than all at once. Complete analysis of the showcase data (370,000 probes) was performed in under an hour on an average desktop PC (Intel Core i5 processor, 4 GB of RAM). For even larger datasets, the Pheno2Geno package is embedded in the xQTL workbench [21,22], allowing for easy parallelization and use of cluster and cloud computing.

**2) Integration with R/qtl.** The package employs well-optimized methods and functions of R/qtl for all the mapping steps as well as filling, estimating and re-estimating maps. Moreover, genetic maps created by Pheno2Geno can be used directly in R/qtl, providing a smooth transition from genetic map creation to QTL mapping.

**3) Strict selection of candidate markers.** Pheno2Geno contains multiple selection steps to filter out candidate markers of low quality e.g. candidate markers affected by multiple QTLs and/or environment. These are then flagged and can easily be excluded from the analysis.

**4) Gene expression phenotypes.** We have demonstrated that Pheno2Geno works on array-based gene expression data. If a gene expression phenotype shows a significant QTL (eQTL) and if this eQTL co-localizes with a probe (a local eQTL), then the derived marker will be placed at the location of that probe. If the QTL does not co-localize with the probe (a distant eQTL), the derived marker will not be placed in the region targeted by the original probe but at the position of the distant eQTL (Figure 2) [23,24].

## Availability and requirements

- **Project name:** Pheno2Geno
- **Project home page:**http://www.pheno2geno.nl
- **Operating system(s):** Any platform for which the R software [25] is implemented, including Microsoft Windows, Mac OS and Linux
- **Programming language:** R
- **Other requirements:** Packages installed in R: qtl, mixtools [11]
- **License:** GNU General Public License version 3
- **Any restrictions to use by non-academics:** None

Analysis software (Additional files 1, 2 and 3), data (Additional file 4) and results (Additional file 5) are provided as supplementary material.

## Limitations

Currently, the Pheno2Geno package can only analyze crosses between diploid inbred strains. If there are no phenotypes with major QTL the method is unable to generate new markers. If there are phenotypes with major QTL their physical location may still be unknown. In the case of gene expression phenotypes the new marker may co-localize with the known physical position of the gene (*cis* effect) or map at a different location (*trans* effect). In the latter case the physical location remains unknown. New markers generated by Pheno2Geno segregate in this cross, but not necessarily in other crosses.

## Additional files

Additional file 1
**Pheno2Geno package.** Contains the R package Pheno2Geno described in this article. The package is distributed under the GNU Public License http://www.gnu.org/. It contains a subset of the data used in this article. The complete dataset is available for download: https://molgenis26.target.rug.nl/downloads/pheno2geno/

Additional file 2
**A compiled version of the Pheno2Geno R package.** Contains the Pheno2Geno package compiled for the Windows operating system.

Additional file 3
**Analysis script** ***A. thaliana*** **.** Contains an R script with the analysis. The figures presented in this paper are produced by this script and the Pheno2Geno package.

Additional file 4
**Original genotypes** ***A. thaliana*** **.** CSV file containing the original genotype and genetic map data.

Additional file 5
**Saturated genotypes** ***A. thaliana*** **.** CSV file containing the improved genotype and genetic map data.

## Abbreviations

AFLP: Amplified fragment length polymorphism
CRAN: Comprehensive R archive network
QTL: Quantitative trait locus
eQTL: expression quantitative trait locus
RIL: Recombinant inbred line
