# Supplementary material for: Pheno2Geno - High-throughput generation of genetic markers and maps from molecular phenotypes for crosses between inbred strains
Source: BMC Bioinformatics. 2015 Feb 19;16(1):51. doi: 10.1186/s12859-015-0475-6 (PMC4339742; doi:10.1186/s12859-015-0475-6)
Supplement: Additional file 2 — A compiled version of the Pheno2Geno R package. Contains the Pheno2Geno package compiled for the Windows operating system. [file 12859_2015_475_MOESM2_ESM.zip › pheno2geno/html/00Index.html]

R: Pheno2Geno - Generating genetic markers and maps from molecular
phenotypes

# Pheno2Geno - Generating genetic markers and maps from molecular phenotypes

---

## Documentation for package ‘pheno2geno’ version 1.3.0

- DESCRIPTION file.

## Help Pages

|  |  |
| --- | --- |
| add.to.population | Add additional data to a population object |
| add.to.populationSub.internal | Add additional data to a population object |
| assignChrToMarkers | Function that assigns a chromosome label to a genetic marker |
| assignMaximum | Create a de novo genetic map from a population object. |
| assignMaximumNoConflicts | Create a de novo genetic map from a population object. |
| avg\_map\_distances | Functions to provide some descriptive statistics on genetic maps |
| coloringMode | Plotting routine for comparison of two genetic maps. |
| create.population | Create a population object |
| cross.denovo | Create a de novo genetic map from a population object. |
| cross.saturate | Saturate an existing genetic map. |
| dataObject | Add additional data to a population object |
| dataType | Add additional data to a population object |
| fake.population | Simulate a population object. |
| find.diff.expressed | Finding differentially expressed genes. |
| find.mixups | Find sample mix-ups |
| generate.biomarkers | Generate discrete biomarkers from the continuous phenotypes |
| groupLabels | Finding differentially expressed genes. |
| logarithmic | plotMarkerDistribution |
| majorityCorrelation | Create a de novo genetic map from a population object. |
| majorityOfMarkers | Create a de novo genetic map from a population object. |
| map\_distances | Functions to provide some descriptive statistics on genetic maps |
| margin | Generate discrete biomarkers from the continuous phenotypes |
| markerPlacementPlot | Plot number of markers selected. |
| markers | Plotting routine for children expression data. |
| markersCorPlot | Plotting correlation between markers on two maps. |
| meanCorrelation | Create a de novo genetic map from a population object. |
| overlapInd | Generate discrete biomarkers from the continuous phenotypes |
| pheno2geno | Pheno2Geno - High-throughput generation of genetic markers and maps from molecular phenotypes |
| phenotypeRow | plotMarkerDistribution |
| plotChildrenExpression | Plotting routine for children expression data. |
| plotMapComparison | Plotting routine for comparison of two genetic maps. |
| plotMarkerDistribution | plotMarkerDistribution |
| plotParentalExpression | Plotting routine for parental expression data. |
| plotRPpval | Visualize the outcome of a Rank product analysis |
| population | Create a population object |
| power.plot | Comparison of power of qtl detection. |
| projectOldMarkers | Plotting routine which shows where markers from original map are located on saturated map. |
| proportion | Generate discrete biomarkers from the continuous phenotypes |
| pull.biomarkers | Extract the detected biomarkers from a population object. |
| qtl.comparison.plot | Comparison of qtl profiles. |
| read.population | Loading genotype and phenotype data |
| reduceChromosomesNumber | Change the number of chromosomes in a cross object |
| removeChromosomes | Change the number of chromosomes in a cross object |
| removeTooSmallChromosomes | Change the number of chromosomes in a cross object |
| reorganizeMarkersWithin | Reorganize markers within cross object. |
| save.gff | Saving gff files. |
| scan.qtls | Scan qtls |
| set.geno.from.cross | Pull genotype from an object of class cross. |
| showRPpval | Visualize the outcome of a Rank product analysis |
| sumMajorityCorrelation | Create a de novo genetic map from a population object. |
| testCross | Test cross object |
| testPopulation | Test population object |
| transformation | Basic functions to do transformation / normalization of phenotypes. |
| write.population | Writes a population object to file. |
